# Radiological and clinical findings of idiopathic myointimal hyperplasia of mesenteric veins: Case report

**DOI:** 10.1097/MD.0000000000027574

**Published:** 2021-10-22

**Authors:** Huanhuan Xie, Xiaopei Xu

**Affiliations:** Department of Radiology, The Second Affiliated Hospital of Zhejiang University School of Medicine, Hangzhou, Zhejiang, China.

**Keywords:** colonic wall thickening, CT features, idiopathic myointimal hyperplasia of the mesenteric veins, ischemic colitis, poor mural enhancement

## Abstract

**Introduction:**

Idiopathic myointimal hyperplasia of mesenteric veins (IMHMV) is an uncommon cause of ischemic bowel disease resulting from the proliferation of smooth muscles in the venous intima. Delayed diagnosis could only be made following the surgical resection due to lack of imaging data, which may lead to bowel severe bleeding, perforation, necrosis, infection, or shock. In previous reports, few cases have provided the detailed pre-operative radiological characteristics of IMHMV. Herein, we are the first to provide the complete clinical course and comprehensive pre-operative radiological data of a 21-year-old female diagnosed with IMHMV.

**Patient concerns:**

A 21-year-old female was admitted to our hospital with bloody diarrhea and abdominal pain. Physical examination revealed tenderness localized to the left lower abdomen. The patient had no prior history of similar symptoms. A computed tomography scan was performed and showed diffuse wall thickening from the rectum to sigmoid colon with poor mural enhancement, multiple ulcers, fat stranding, and free fluid. The arterial phase images demonstrated many tortuous pericolic arteries and submucosal pseudoaneurysm.

**Intervention:**

Conservative treatment including empirical antibiotics, Mesalazine, and methylprednisolone sodium succinate were administrated to relief the symptoms. However, the diarrhea and abdominal pain worsened. An emergency surgery was arranged and total proctocolectomy with ileal pouchanal anastomosis with ileostomy was performed.

**Diagnosis:**

Macroscopic and histopathological examinations of the excised specimen showed ischemic colitis. Elastica van Gieson staining revealed extensive myointimal hyperplasia and confirmed the diagnosis of IMHMV.

**Outcomes:**

During the 2-year follow-up period, no additional medical management was needed. The patient was well and surveillance colonoscopy showed normal colon and anastomosis.

**Conclusion:**

Pre-operative computed tomography with imaging features including pronounced continuous concentric thickening colonic wall with poor enhancement and enlarged tortuous pericolic arteries could specifically facilitate the speedy diagnosis of IMHMV.

## 1 Introduction

Idiopathic myointimal hyperplasia of mesenteric veins (IMHMV) is a rare condition which poses diagnostic challenge to pathologists, radiologists, and clinicians. It has often been misdiagnosed as inflammatory bowel disease (IBD) or ischemic colitis due to their similar clinical manifestations. IMHMV primarily affects the rectosigmoid colon of middle-aged men, while the small intestine^[1]^ is only involved in very few patients. Most patients suffer from persistent abdominal pain and hematochezia,^[2]^ while some patients present with weight loss and small bowel obstruction.^[3]^ The pathogenesis of the myointimal hyperplasia in mesenteric veins is still unknown. The more widely accepted hypothesis is that IMHMV stems from an acquired vascular or hemodynamic etiology, which believed that the traumatic injury of sigmoid mesocolon secondary to torsion or stretching could lead to arteriovenous fistulization and eventually myointimal hyperplasia of the mesenteric veins.^[4]^ IMHMV usually requires complete surgical resection rather than medical treatment like IBD does. Delayed diagnosis may lead to bowel severe bleeding, perforation, necrosis, infection, or shock. In previous reports, few cases have provided the detailed pre-operative radiological characteristics of IMHMV. Here, we describe the detailed computed tomography (CT) imaging features and clinical course of a 21-year-old female. A review of the pertinent literatures is also given.

## 2 Case presentation

### 2.1 Clinical history

**Figure 1:**
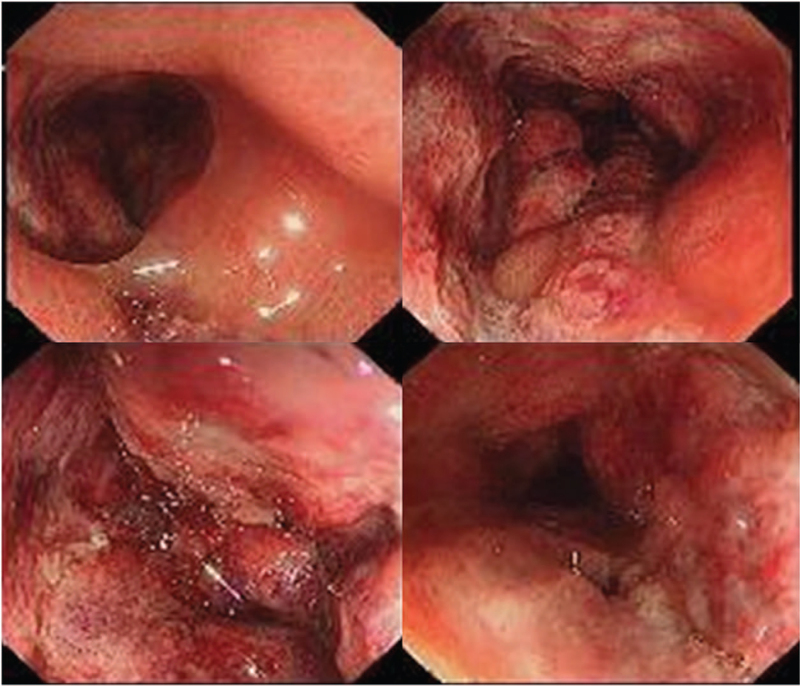
Colonoscopy revealed diffuse severe edema and circumferential ulceration with exudates in the distal descending colon to the rectum. The mucosa was congested, edematous, and erythematous. The submucosal vessels were invisible and easily bleeding.

A 21-year-old female was admitted to our hospital with abdominal pain, tenesmus, and bloody diarrhea for more than 10 days. Physical examination revealed tenderness localized to the left lower abdomen. The patient had no prior history of similar symptoms and no prior history of surgery, trauma, or connective tissue disorders. On admission, initial laboratory examinations revealed an elevated level of white blood cell count (18,700/mm^3^), C-reactive protein (14.6 mg/L), D-dimer (6820 μg/L), and carbohydrate antigen 125 (CA125, 60.6 U/mL). Colonoscopy at initial workup revealed edematous wall thickening, hyperemia and shallow ulceration with continuous involvement from rectum to sigmoid colon. The friable mucosa was difficult to identify and was easily bleeding (Fig. 1). No obvious abnormalities in transverse colon, ascending colon, and ileocecum was identified.

### 2.2 Radiological findings

**Figure 2:**
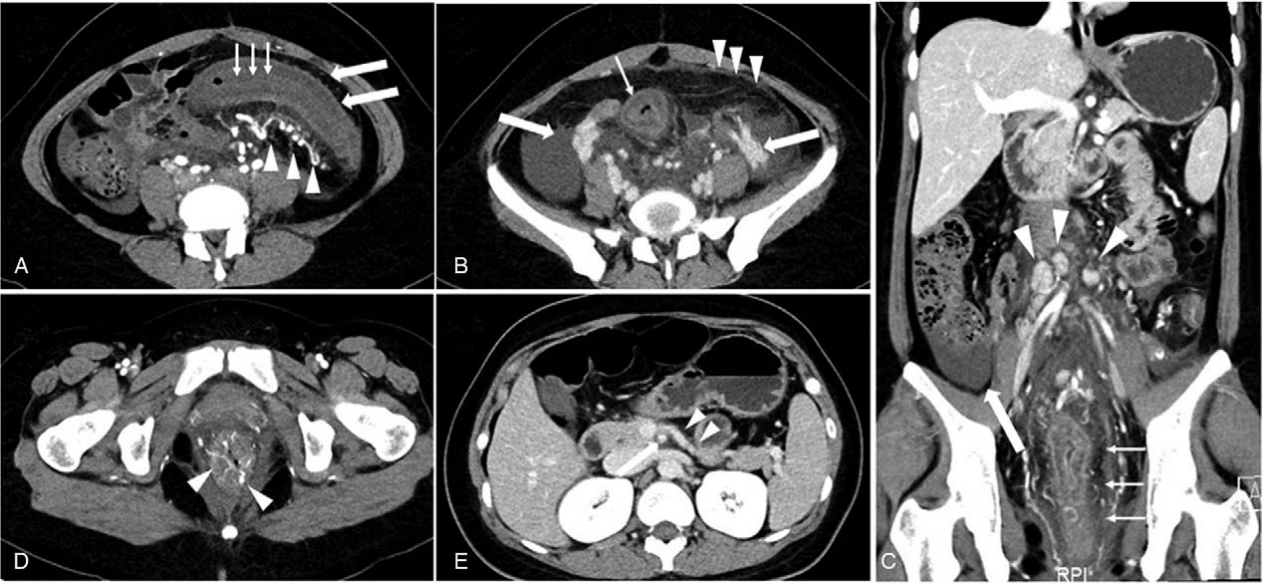
Abdominopelvic CT findings of the IMHMV. Axial image of arterial phase (A) demonstrated the continuous concentric thick, edematous colonic wall (thick arrows) with poor enhancement and ulceration (arrows) of colonic wall. The prominent pericolic arteries (arrowheads) with pseudoaneurysm are the specific signs of IMHMV. Axial image of portal phases (B) showed the fat stranding, omental cake sign (arrowheads), and dilated ovarian veins (thick arrows). Oblique coronal reformatted image (C) of portal phase demonstrated severe rectum wall thickening with mural stratification, pericolic fat infiltration (arrows) and ascites (thick arrows). Many retroperitoneal enlarged lymph nodes were showed (arrowheads). Pseudoaneurysm (D) in the submucosa might be responsible for the bloody diarrhea. Axial delayed phase image (E) demonstrated the patent inferior mesenteric vein (arrowheads), superior mesenteric artery and superior mesenteric vein (thick arrows). CT = computed tomography, IMHMV = idiopathic myointimal hyperplasia of mesenteric veins.

Multiphase contrast-enhanced abdominopelvic CT were performed on the second day of admission. The CT imaging showed a long segment of marked wall thickening extending from the sigmoid colon to the distal rectum with poor mural enhancement, multiple ulcers, pericolic fat stranding, and free fluid. The colonic wall thickened to 1.6 cm and the marked submucosal edema caused the target sign of colonic wall. Thickening of the omentum was also noted while multiple enlarged lymph nodes could be seen in the retroperitoneum (Fig. 2A–C). The arterial phase images demonstrated the inferior mesentery artery with enlarged tortuous pericolic arteries and submucosal pseudoaneurysm (Fig. 2A, D). The portal phase image showed that the inferior mesenteric vein (IMV) was patent without filling defects or luminal irregularities (Fig. 2E). However, the ovarian veins were tortuous and dilated on the delayed phase images (Fig. 2B).

### 2.3 Treatment

Based on the clinical symptoms, acute IBD was clinically suspected, and the patient was treated with empirical antibiotics (Sulperazon). However, the diarrhea and abdominal pain worsened overnight and resulted in sleep disturbance of the patient. Mesalazine and methylprednisolone sodium succinate were administrated after 4 days though did not offer symptomatic relief. Ten days after admission, the patient developed fever, massive hematochezia with decreased hemoglobin (from 123–57 g/L), and elevated white blood cell count and C-reactive protein (30,500/mm^3^ and 63.5 mg/L, respectively). The patient went into shock on her way to the second CT scan and was transferred to the emergency room immediately.

### 2.4 Intra-operative findings and pathological examination results

The clinician explained the necessity of surgical resection to the patient. Total proctocolectomy with ileal pouchanal anastomosis with ileostomy was performed on the same day. Intra-operative exploration revealed multiple ulcerative lesions in the colon and rectum, with diffuse bleeding and local necrosis and perforations. Microscopically, mucosal ulcer, sub-mucosal edema with hemorrhage, and chronic serositis with fat necrosis were observed in the involved colon. Importantly, fibrous intimal thickening and luminal occlusion of mesentery and subserosal vascular could be observed, while there were no findings of surrounding venulitis or thrombi within veins. These findings were consistent with IMHMV. Additionally, Elastica van Gieson staining confirmed the presence of elastic fiber at the site of the thickened venous intima and the final diagnosis has been confirmed as IMHMV.

### 2.5 Postoperative course

The patient was discharged 10 days after the surgery with no postoperative complications. At 4 months after the operation, the patient had a swift recovery without any additional medication. During the 2-years follow-up period, there was no evidence of disease recurrence. Postoperative contrast-enhanced abdominopelvic CT imaging indicated no abnormality.

## 3 Discussion and conclusions

In 1991, Genta and Haggitt^[5]^ were the first to describe 4 patients with segmental ischemic colitis caused by idiopathic myointimal hyperplasia in the small mesenteric veins. IMHMV is a rare and poorly understood cause of non-thrombotic, non-inflammatory mesenteric venous occlusion which affects the rectum and sigmoid colon. It is often misdiagnosed as IBD due to their similar clinical symptoms such as abdominal pain and bloody diarrhea. These patients often suffer from frequently prolonged clinical course and are placed on high doses of immunosuppressant which hindered the proper treatment.^[6]^ IMHMV is rarely recognized in mucosal biopsies. Histologically, the distinctive pathognomonic feature includes non-thrombotic, non-inflammatory occlusion of the involved vessels secondary to extensive myointimal hyperplasia of the mesenteric veins in submucosa, adventitia, and mesocolon.^[7]^ Venous myointimal hyperplasia without surrounding venulitis and arteritis is the key feature in the diagnosis of IMHMV. In addition, IMHMV has severe complications such as enterobrosis or severe internal bleeding which require emergency surgery.^[4]^ Segmental resection is curative and there have not been any reports of the postoperative disease recurrence.^[8,9]^

The distinctive pre-operative CT features of IMHMV have yet to be fully described. So far, there have been 7 case reports about the expression of CT in IMHMV (Table 1). Together with our present case, the sigmoid colon was involved in 6 patients and rectum in 5 patients. There were 2 cases involving the descending colon or transverse colon. Only 1 patient had a focal lesion involving the ileum. Thus, the involvement almost confined to the rectosigmoid colon. To the best of our knowledge, IMHMV was only reported in the small bowel of 6 cases.^[3,10–14]^ Two of 7 cases were focal lesion and 1 case had small bowel obstruction.^[3,15]^ The focal lesion suspicious of a malignancy was easily misdiagnosed. Four out of 7 cases showed pericolic vessels with aneurysmal change or vascular congestion. All cases showed the distinct wall thickening and fat stranding.

**Table 1:** Summary of radiological findings of IMHMV in previously reported cases.

Case	Author	Year	Age/gender	Clinical presentation	Location	Imaging modalities	CT findings
1	Sahara et al^[20]^	2015	76/M	Diarrhea, abdominal pain	Sigmoid colon, rectum	CECT	Wall thickening, severe edema, and the fat stranding
2	Yang et al^[15]^	2016	44/M	Diarrhea, abdominal pain	Recto-sigmoid junction	CECT	Focal wall thickening, fat stranding
3	Garcia-Castellanos et al^[17]^	2011	32/F	Diarrhea with blood and mucus, abdominal pain	Sigmoid colon, rectum	CECT/angiography	Wall thickening, fat stranding, hypertrophic and collateral vessels
4	Ansari et al^[18]^	2021	63/M	Non-bloody diarrhea, abdominal pain	Distal transverse colon to sigmoid	CECT	Wall thickening, serosal irregularity, pericolic inflammation change with mesocolic vascular congestion and hyperemia, free fluid
5	Martin et al^[1]^	2019	63/M	From watery diarrhea to progressive bloody diarrhea, fecal incontinence, weight loss	Distal descending colon to the rectum	CECT/angiography	Contiguous concentric wall thickening, fat stranding, engorgement of the vessels
6	Yun et al^[2]^	2016	64/M	Diarrhea, left lower abdominal pain,	Descending colon, sigmoid, rectum	CECT/angiography	Active bleeding, wall thickening, mural stratification, poor bowel wall enhancement and pericolic fat infiltration, pericolic veins with aneurysmal change
7	Yamada et al^[3]^	2021	81/F	Abdominal pain, nausea, and vomiting	Ileum	CECT/barium X-ray series	Focal wall thickening, stenosis of the terminal ileum, bowel obstruction

In addition, pronounced non-segmental, concentric, colonic wall thickening, which is consistent with the ischemic changes, is also a prominent radiological appearance of IMHMV. The marked submucosal edema caused the colonic wall a target sign. However, bowel wall thickening is also the least specific CT finding in cases of bowel ischemia, since it may be observed in a variety of non-ischemic conditions affecting the small or large bowel.^[16]^ Another common sign which discriminates IMHVM from IBD is the poor mural enhancement. The intestinal wall edema and poor enhancement usually resulted from the hemodynamic changes and increased blood pressure due to the proliferation of smooth muscles in the venous intima. Histologically, the mesenteric veins in IMHMV closely resemble those seen in failed cardiac saphenous vein bypass grafts, consistent with a secondary ‘arterialization’ effect caused by greatly increased pressure within affected veins.^[7]^ Abu-Alfa et al^[7]^ hypothesized that the venous myointimal hyperplasia in IMHMV is caused by the increased intraluminal pressure due to an acquired segmental arteriovenous fistulization. We recognized that pre-operative contrast-enhanced CT showed many dilating peripheral arteries in the colonic region with edematous wall thickening. These findings may specifically support the diagnosis of IMHMV. Yun et al^[2]^ and García-Castellanos^[17]^ et al have reported a similar case demonstrating prominent pericolic veins with aneurysmal change and occlusion of the distal IMV using CT or angiography. Ansari et al^[18]^ also reported mesocolic vascular congestion and hyperemia signs. Chiang et al^[19]^ reported no demonstrable IMV is observed during venous-phase angiography and no arteriovenous fistula was found. However, our case demonstrated the IMV was patent. Thus, we concluded that IMV may be demonstrable in early stage of the disease. As the disease progresses, IMV may be occluded and arteriovenous fistula occur. All these reports showed no filling defects or luminal irregularities in the inferior mesentery artery and IMV. This was distinct from the mesenteric venous thrombosis cause of ischemic bowel disease. Therefore, we should examine angiography to investigate for venous occlusive diseases.^[20]^ Previous literature reported that severe bloody stool was common. This symptom may lead to shock and even death threatening in patients. We recognize that the presence of multiple aneurysmal arteries in the submucosa is an important cause of bloody stool.

Ulceration and inflammatory exudates may be seen later in the disease process.^[4]^ Our case indicated the ulceration of IMHMV was widespread and transmural. Severe bleeding and perforation could occur finally. Meanwhile, we found that the fat stranding was more diffuse, more severe in IMHMV than IBD. Our case showed the omental cake sign and showed a large amount of ascites.

The differential diagnosis of IMHMV includes IBD especially ulcerative colitis (UC), other ischemic bowel diseases such as mesenteric vasculitis and mesenteric venous thrombosis, and mesenteric inflammatory veno-occlusive disease (MIVOD). The most predilection sites and pronounced non-segmental wall thickening were similar to UC. The distinction between the 2 disease always was difficult. CT findings of UC include hyperenhancement and colorectal narrowing, widening of presacral space which differs from IMHMV. Contiguous thickening of the colon to the rectum was the distinguishing sign with Crohn disease which always manifested as discontinuous and eccentric thickening of the intestinal wall. The site of involvement in IMHMV was distinct from other ischemic bowel diseases such as mesenteric vasculitis. One of the most characteristic features of mesenteric vasculitis is the tendency to involve both the jejunum and the ileum, as well as both the small and large intestine.^[21]^ Mesenteric venous thrombosis can be caused by various reasons. Thrombosis can be confidently detected with CT, even in the peripheral branches in nowadays. The CT features of MIVOD are similar to IMHMV, except the site of involvement. MIVOD has been reported to occur in all colonic segments as well as in the small bowel.^[22]^ The pre-operative diagnosis of focal lesions was challenging. Sherman et al^[23]^ reported that focal myointimal hyperplasia of mesenteric veins were associated with pre-resection trauma to the involved bowel segment. Thus, we need to be differentiated from colon cancer combining the clinical history and serum tumor markers.

In summary, we described the distinguishing CT features and complete clinical course of a rare case of IMHMV. Despite its rarity, we found that the characteristic radiological features of IMHMV include: typical sites of involvement including descending colon, sigmoid, and rectum; pronounced continuous concentric thick, edematous colonic wall with poor enhancement; no mesenteric venous thrombosis or occlusion in the IMV; many dilating peripheral arteries and pseudoaneurysm; ulceration and fat stranding. Although the diagnosis of IMHMV was difficult in patients with abdominal pain and bloody diarrhea, these radiological features may contribute to the pre-operative diagnosis of IMHMV. Radiologists should alert the clinicians with the possibility of IMHMV when noticing these imaging features, so as to perform the surgery in time and reducing diagnostic delays. Prompt operation can reduce patients’ suffering and improve their quality of life.

## Author contributions

Hh X analyzed and interpreted the patient data and was a major contributor in writing the manuscript. Xp X designed the study and edited the manuscript. All authors read and approved the final manuscript.

**Investigation:** Huanhuan Xie.

**Supervision:** Xiaopei Xu.

**Writing – original draft:** Huanhuan Xie.

**Writing – review & editing:** Xiaopei Xu.
